# High Expression Levels of the Long Non-Coding RNAs Lnc-IRF2-3 and Lnc-KIAA1755-4 Are Markers of Poor Prognosis in Chronic Lymphocytic Leukemia

**DOI:** 10.3390/ijms26031153

**Published:** 2025-01-29

**Authors:** Natasa Tosic, Kristina Tomic Vujovic, Vojin Vukovic, Nikola Kotur, Biljana Stankovic, Irena Marjanovic, Darko Antic, Sofija Sarac, Tamara Bibic, Jelena Ivanovic, Branka Zukic, Teodora Karan-Djurasevic

**Affiliations:** 1 Institute of Molecular Genetics and Genetic Engineering, University of Belgrade, Vojvode Stepe 444a, 11042 Belgrade, Serbia; natasa.tosic@imgge.bg.ac.rs (N.T.); nikola.kotur@imgge.bg.ac.rs (N.K.); biljana.stankovic@imgge.bg.ac.rs (B.S.); irena.marjanovic@imgge.bg.ac.rs (I.M.); branka.zukic@imgge.bg.ac.rs (B.Z.); 2 Clinic for Hematology, University Clinical Center of Serbia, 11000 Belgrade, Serbia; kristinat992@gmail.com (K.T.V.); vojinvukovic@yahoo.com (V.V.); darko.antic1510976@gmail.com (D.A.); sofijasarac@gmail.com (S.S.); tmedic83@gmail.com (T.B.); jivanovic09@gmail.com (J.I.); 3 School of Medicine, University of Belgrade, 11000 Belgrade, Serbia

**Keywords:** chronic lymphocytic leukemia, long non-coding RNA, lnc-IRF2-3, lnc-KIAA1755-4, expression, prognosis

## Abstract

Long non-coding RNAs (lncRNAs) play complex roles at multiple levels of gene regulation, thus modulating key cellular processes involved in the pathogenesis and progression of cancer. Aberrant expression of lncRNAs has been reported in various malignancies, including chronic lymphocytic leukemia (CLL). We investigated the expression of lnc-IRF2-3 and lnc-KIAA1755-4 in peripheral blood mononuclear cells of 112 previously untreated CLL patients by quantitative reverse-transcriptase polymerase chain reaction. Both lncRNAs were found to be overexpressed in CLL samples in comparison to healthy controls, and their high levels were associated with adverse clinico-biological characteristics of patients at diagnosis. High lnc-IRF2-3 expression was associated with high leukocyte and lymphocyte counts, high β2-microglobulin, advanced Binet stage, unfavorable cytogenetics, CD38-positivity and *IGHV*-unmutated status. Regarding lnc-KIAA1755-4, its high expression was associated with high leukocyte count, lymphocyte count, β2-microglobulin, lactate dehydrogenase and low hemoglobin, as well as with *IGHV*-unmutated status. In addition, we observed shorter time to first treatment and overall survival of patients expressing high levels of both lncRNAs in comparison to low-expressing patients. In summary, our study showed that high lnc-IRF2-3 and lnc-KIAA1755-4 expression at diagnosis predicts poor survival in CLL. The mechanisms of their upregulation, as well as their specific targets in CLL cells, remain to be elucidated.

## 1. Introduction

Chronic lymphocytic leukemia (CLL) is a hematological neoplasm characterized by the clonal expansion of mature B-lymphocytes co-expressing CD5 with typical B-cell surface antigens, which gradually accumulate in the blood, bone marrow and secondary lymphoid organs [1]. The clinical course of CLL is very heterogeneous, ranging from indolent for years, even decades, to rapidly progressive soon after the onset. In the majority of patients, though, the aggressiveness of CLL lies somewhere in between these two extremes, which has prompted an ongoing search for markers that can predict the clinical behavior of the disease in its early stages [1,2,3]. The pathogenesis and progression of CLL result from the complex interplay between extrinsic factors from the tissue microenvironment and intrinsic genetic abnormalities of CLL cells. The latter encompass recurrent cytogenetic aberrations (del13q14, trisomy 12, del11q22-23, del17p13, complex caryotype), mutations and dysregulated expression of protein-coding genes (*NOTCH1*, *SF3B1*, *XPO1*, *BIRC3*, *NFKBIE*, *TP53*, *ATM*, *POT1*, *BCL2*, *BCL-XL*, *MCL1*, etc.), but also the alterations of non-coding RNA species, including long non-coding RNAs [4,5,6,7,8].

Long non-coding RNAs (lncRNAs) are defined as transcripts longer than 200 nucleotides that are not translated into proteins. LncRNAs can be intergenic, antisense, or partially overlapping exons, introns or regulatory regions of protein-coding genes, depending on the positions of their loci. LncRNAs exhibit some similarities with mRNAs, such as transcription by RNA polymerase II, 5′-capping, 3′-polyadenylation, splicing and nuclear localization. On the other hand, they are extremely cell type- and differentiation stage-specific and are generally expressed at significantly lower rates than mRNAs [9]. LncRNAs directly interact with mRNAs, non-coding RNAs, DNA and proteins, to regulate gene expression in cis and in trans at multiple levels: epigenetic, transcriptional, post-transcriptional, translational and post-translational. LncRNAs modulate transcription by forming DNA–RNA hybrids recognized by chromatin modifiers and transcription factors, or acting as guides, decoys or scaffolds for proteins involved in epigenetic modifications and chromatin remodeling. Furthermore, lncRNAs directly regulate transcription by recruiting transcription factors to target gene promoters or sequestering them from promoters, or by affecting neighboring genes’ transcription. By forming ribonucleoprotein complexes, lncRNAs scaffold nuclear condensates which regulate transcription and alternative splicing. LncRNAs also regulate mRNA editing, stability, decay, translation, post-translational modifications and protein localization by interacting with RNA and proteins. LncRNAs can serve as miRNA and siRNA precursors or can sponge miRNAs from their target mRNAs [9,10,11]. By employing all these mechanisms of gene regulation, lncRNAs modulate a multitude of processes in a way specific to a given cellular context.

LncRNAs have emerged as important players in both normal and malignant B-cell biology. The expression of certain lncRNAs is tightly regulated during normal B-cell development and is restricted to specific stages of differentiation and B-cell subsets. In addition, lncRNAs are involved in the regulation of key cancer-related processes (DNA-damage response, cell cycle progression, proliferation, apoptosis, differentiation, communication with the microenvironment), exerting both oncogenic and tumor-suppressive functions, and their dysregulation has been implicated in the pathogenesis of various B-cell malignancies [12,13,14,15,16]. In B-cell lymphomas and leukemias, lncRNAs regulate cell death through modulation of Bcl2 family-mediated apoptosis (DLEU1/2, HULC), Fas-mediated apoptosis (FAS-AS1), or by interactions with the p53 pathway during DNA damage response (lincRNA-p21, NEAT1) [17,18,19,20,21,22]. LncRNAs also modulate other signaling pathways related to apoptosis and proliferation, e.g., PI3K/Akt (HOTAIR), MAPK/Erk (PANDA), NF-κB (DLEU1/2), Wnt/β-catenin (OR3A4, FIRRE, SMAD5-AS1), Il6 (lnc-TOMM7-1), and regulate the expression of cell cycle checkpoint proteins (MALAT1, lincRNA-p21, LUNAR1, MINCR) [23,24,25,26,27,28,29,30,31,32,33]. Some lncRNA participate in proliferative pathways induced by Myc, a proto-oncogene frequently activated in aggressive B-cell lymphomas and leukemias (FIRRE, DANCR, GAS5, RMRP, PVT1) [27,34,35,36,37]. Another mechanism employed by multiple lncRNAs in promoting cell growth is their binding to the EZH2/PRC2 complex, thus modulating the transcription of p21 and p27 (MALAT1, ROR1-AS1) [30,38]. In addition, lncRNAs are associated with genomic stability and telomere maintenance (treRNA, TERRA) [39,40]. Regarding interactions with the microenvironment, some lncRNAs were shown to be deregulated during these interactions or to modulate B-cell responses to stimuli that trigger B-cell receptor (BCR) and other signaling pathways (BCALM, LINC00461, XIST, LINC00152) [41,42,43,44].

In CLL, aberrant expression of a number of lncRNAs was reported (e.g., DLEU1/2, LincRNA-p21, NEAT1, MIAT, CRNDE, TRERNA1, lnc-TOMM7-1, GAS5, LEF1-AS1, ZNF667-AS1, BM742401, MALAT1, etc.), some of which were shown to be associated with the course of the disease, as well as with the clinical and biological prognostic markers [7,8,45,46,47,48]. Moreover, the pathogenic mechanisms of some of the recurrent genetic abnormalities in CLL have been linked, at least in part, to their effect on lncRNAs. A typical example is del13q14, whose minimal deleted region contains the lncRNA DLEU2 gene and the first exon of the DLEU1 gene. DLEU2 is a host gene of miR-15a and miR-16-1 which downregulate the G1 phase cyclins, repress Bcl2 at the post-transcriptional level and modulate NF-κB signaling. Thus, functional loss of miR-15a and miR-16-1 has a pro-proliferative and anti-apoptotic effect and plays a crucial role in the pathogenesis of CLL [17,25,49,50]. Another example is *TP53* mutations; it was demonstrated that lincRNA-p21 and NEAT1 are targets of the p53 protein and that they induce apoptosis upon DNA damage in primary CLL cells, but only in the *TP53* wild-type setting [20]. Furthermore, extrinsic factors from the lymph node microenvironment also affect the expression of some lncRNA. For example, in CLL cells upregulation of LINC00152 was observed upon stimulation of TLR9 [44]. For the majority of lncRNAs dysregulated in CLL, though, the mechanisms leading to their abnormal expression, as well as their modes of action in CLL B-cells, are still elusive.

In a study conducted by Ronchetti et al., a comprehensive lncRNA expression profiling was performed in a cohort of newly diagnosed, early-stage CLL patients, which revealed a set of lncRNAs that are deregulated in CLL cells in comparison to normal B-cell counterparts. Among those differentially expressed lncRNAs, some were upregulated and others downregulated, and some of them were recurrently associated with the status of several prognostic markers (somatic hypermutational (SHM) status of immunoglobulin heavy-variable (*IGHV*) genes, chromosomal aberrations, *NOTCH1* mutations, CD38 and ZAP70 expression). Ronchetti et al. also identified a set of lncRNAs that were associated with the progression of CLL, namely with the time to first treatment (TTFT); based on the expression of two lncRNAs which exerted the highest predictive value for TTFT, lnc-IRF2-3 and lnc-KIAA1755-4, the authors proposed the “2-lncRNA risk model” which stratifies CLL patients into three prognostic groups: high-risk group, characterized by high expression of both lncRNAs, intermediate-risk group with discordant expression of the two lncRNAs, and low-risk group with the concomitant low expression of both lncRNAs [29]. Upregulation of lnc-IRF2-3 in CLL was also reported by El-Khazragy et al., as well as the association of its high expression level with adverse prognostic markers and shorter TTFT and overall survival (OS) [51]. However, lnc-KIAA1755-4 has not been further investigated in CLL.

The aim of our study was to analyze the expression pattern of lnc-IRF2-3 and lnc-KIAA1755-4 in a cohort of unselected CLL patients before the initiation of treatment and to assess the association of their individual expression levels with clinical and laboratory features, established prognostic markers and survival. In addition, by analyzing the combined expression of lnc-IRF2-3 and lnc-KIAA1755-4, we aimed to test the above-mentioned “2-lncRNA risk model” in a heterogeneous CLL cohort.

## 2. Results

### 2.1. Characteristics of the Cohort

This retrospective study involved 112 unselected CLL patients and 21 healthy control individuals. The CLL group consisted of 79 men and 33 women (male/female ratio = 2.4), with a median age of 59 years at diagnosis (range: 33–80). Demographic, clinical and biological features of CLL patients are summarized in Table 1. The control group consisted of 15 men and 6 women (male/female ratio = 2.5), aged 63–85 years (median = 71).

During the median follow-up time of 73.5 months (range: 4–360), 59 patients died (52.7%), 43 patients were still alive (38.4%), and 10 patients were lost (8.9%). The majority of patients in our cohort (89/112; 79.5%) received treatment (87 patients had chemo(immuno)therapy and 2 patients had ibrutinib), while 23 patients (20.5%) remained untreated during the follow-up period. Median TTFT was 22 months (95% confidence interval (CI) = 8.503–35.497; range: 0–348). In the first therapy line, the majority of patients (67/89, 75.3%) were treated with the fludarabine+cyclophosphamide (FC) based regimen; 65.7% (44 patients) with FC and 34.3% (23 patients) with fludarabine+cyclophosphamide+rituximab (FCR) (Supplementary Table S1). The median progression-free survival after the first therapy line was 29.5 months in patients treated with FC and 48 months in patients treated with FCR, while the median OS was 72.5 months for FC- and 74 months for FCR-treated patients (which was slightly lower than in the CLL8 trial, but comparable with other real-world studies) [52,53,54]. Two patients in our cohort (1.8%) experienced Richter transformation, both of them after the first therapy line. In the whole cohort, the median OS was 92 months (95% CI = 75.834–108.166), with a 5-year survival rate of 76.4%.

Stratification of patients according to the International Prognostic Index for CLL (CLL-IPI) was performed in 53 patients for whom we possessed the necessary data, namely age at diagnosis, Binet stage, serum β2-microglobulin, *IGHV* SHM status and *TP53* mutations and/or del17p13 (in 15/53 patients CLL-IPI scores were calculated without the information about TP53 mutational status (Table 1).

### 2.2. Association of Lnc-IRF2-3 and Lnc-KIAA1755-4 Expression with the Clinical Variables and Prognostic Markers

**Figure 1:**
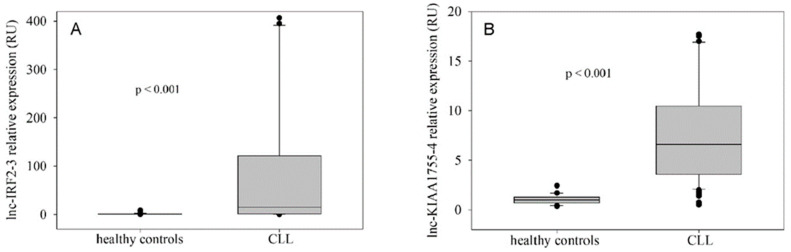
**Expression of lnc-IRF2-3 (A) and lnc-KIAA1755-4 (B) in CLL and control group.** Both lncRNAs were found to be overexpressed in mononuclear cells of CLL patients in comparison to healthy controls. (**A**) CLL (n = 112): median 14.5 RU; range 0.04–1705.53; 25th percentile 1.19 RU; 75th percentile 121.66 RU. Healthy controls (n = 21): median 1 RU; range 0.21–7.81 RU; 25th percentile 0.61 RU; 75th percentile 1.28 RU. (*p* < 0.001, Mann–Whitney Rank Sum Test). (**B**) CLL (n = 112): median 6.6 RU; range 0.52–61.23 RU; 25th percentile 3.58 RU; 75th percentile 10.48 RU. Healthy controls (n = 21): median 1 RU; range 0.36–2.44 RU; 25th percentile 0.68 RU; 75th percentile 1.3 RU. (*p* < 0.001, Mann–Whitney Rank Sum Test). Abbreviations: RU = relative units.

The expression of lnc-IRF2-3 and lnc-KIAA1755-4 in peripheral blood mononuclear cells (PBMNC), measured by quantitative reverse-transcriptase polymerase chain reaction (qRT-PCR), was significantly higher in CLL patients in comparison to healthy controls (*p* < 0.001, Mann–Whitney rank-sum test). The extent of lnc-IRF2-3 upregulation was more prominent than for lnc-KIAA1755-4, and exerted higher patient-to-patient variability (Figure 1). In addition, the expression levels of lnc-IRF2-3 and lnc-KIAA1755-4 were positively correlated in the patient group (r = 0.333, *p* < 0.001, Spearman rank-order correlation test), while in the control group, this correlation was not detected.

In order to investigate the association between lnc-IRF2-3 and lnc-KIAA1755-4 expression and the disease-related variables, median expression levels were used as a cut-off to divide the CLL cohort into lnc-IRF2-3 low- and lnc-IRF2-3 high-expressing groups, as well as lnc-KIAA1755-4 low- and lnc-KIAA1755-4 high-expressing groups. The observed associations are presented in Table 1.

**Figure 2:**
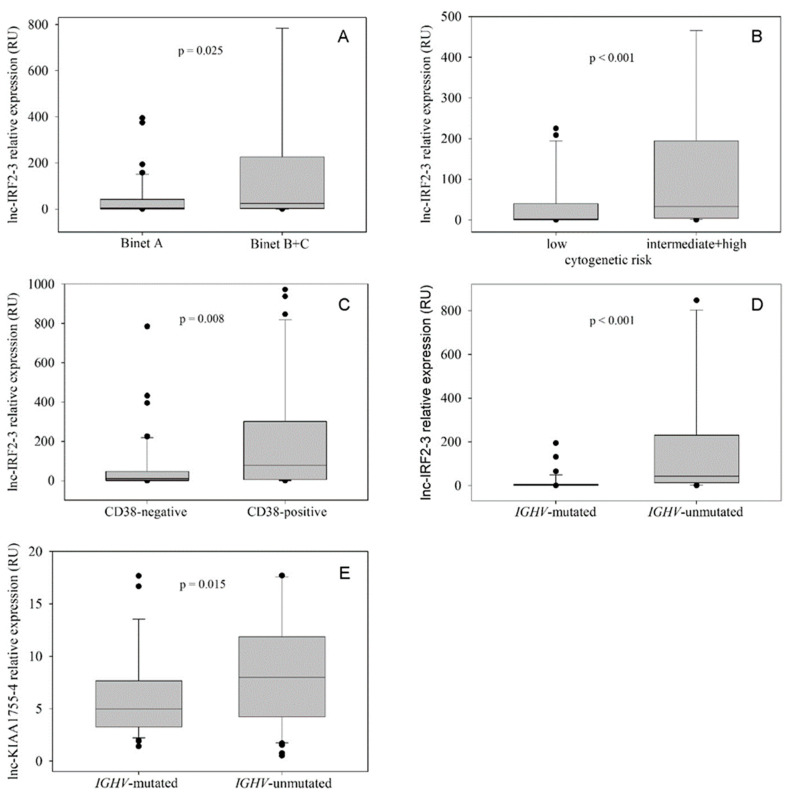
**Expression of lnc-IRF2-3 and lnc-KIAA1755-4 in different prognostic subgroups.** High expression levels of the investigated lncRNAs were associated with the adverse prognostic markers. (**A**) **Lnc-IRF2-3 and Binet stage.** Binet A (n = 52): median 4.36 RU; 25th percentile 0.95 RU; 75th percentile 42.36 RU. Binet B + C (n = 59): median 24.15 RU; 25th percentile 2.54 RU; 75th percentile 225.03 RU. (*p* = 0.025, Mann–Whitney Rank Sum Test). (**B**) **Lnc-IRF2-3 and cytogenetic risk.** Favorable (n = 31): median 1.88 RU; 25th percentile 0.61 RU; 75th percentile 39.62 RU. Intermediate+unfavorable (n = 66): median 32.73 RU; 25th percentile 4.21 RU; 75th percentile 193.88 RU. (*p* < 0.001, Mann–Whitney Rank Sum Test). (**C**) **Lnc-IRF2-3 and CD38 status.** CD38-negative (n = 71): median 8.99 RU; 25th percentile 1.15 RU; 75th percentile 47.18 RU. CD38-positive (n = 30): median 78.87 RU; 25th percentile 4.13 RU; 75th percentile 301.04 RU. (*p* = 0.008, Mann–Whitney Rank Sum Test). (**D**) **Lnc-IRF2-3 and *IGHV* SHM status.** *IGHV*-mutated (n = 46): median 1.14 RU; 25th percentile 0.42 RU; 75th percentile 4.78 RU. *IGHV*-unmutated (n = 66): median 42.98 RU; 25th percentile 12.67 RU; 75th percentile 230.24 RU. (*p* < 0.001, Mann–Whitney Rank Sum Test). (**E**) **Lnc-KIAA1755-4 and *IGHV* SHM status.** *IGHV*-mutated (n = 46): median 4.96 RU; 25th percentile 3.25 RU; 75th percentile 7.66 RU. *IGHV*-unmutated (n = 66): median 8.00 RU; 25th percentile 4.22 RU; 75th percentile 11.86 RU. (*p* = 0.015, Mann–Whitney Rank Sum Test).

When analyzing the clinical variables evidenced at diagnosis, we detected a positive correlation of lnc-IRF2-3 expression with white blood cell count (WBC), lymphocyte count and serum β2-microglobulin (r = 0.285, *p* = 0.005; r = 0.303, *p* = 0.004 and r = 0.385, *p* = 0.003, respectively; Spearman rank-order correlation test). On the other hand, lnc-IRF2-3 expression was not associated with age, sex, hemoglobin (Hb) level, platelet count and serum lactate dehydrogenase (LDH). In addition, we observed a significantly different distribution of clinical Binet stages between the lnc-IRF2-3^low^ and lnc-IRF2-3^high^ groups; patients belonging to early (Binet A) stage were predominant in the lnc-IRF2-3^low^ group, as opposed to the lnc-IRF2-3^high^ group, in which predominated patents in more advanced clinical stages (Binet B and C) (*p* = 0.037, Fisher exact test). Grouping Binet B and Binet C patients together showed that lnc-IRF2-3 expression was significantly higher in the Binet B+C group than in the group composed of Binet A patients (*p* = 0.025, Mann–Whitney rank-sum test) (Figure 2A).

Similarly to Binet stages, patients belonging to three cytogenetic risk groups (favorable—del13q as a sole chromosomal abnormality; intermediate—no aberrations, trisomy 12q; unfavorable—del11q22-23, del17p13) were also differently distributed between the lnc-IRF2-3^low^ and lnc-IRF2-3^high^ groups, with a significantly higher frequency of patients with intermediate and unfavorable cytogenetic risk in the IRF2-3^high^ group (*p* = 0.004, Fisher exact test). By grouping the patients with intermediate and unfavorable cytogenetics together, we observed higher lnc-IRF2-3 expression in this group when compared to patients with low-risk cytogenetics (*p* < 0.001, Mann–Whitney rank-sum test) (Figure 2B). Regarding other biological prognostic markers, we detected higher lnc-IRF2-3 expression in CD38-positive vs. CD38-negative patients (*p* = 0.008, Mann–Whitney rank-sum test) (Figure 2C), as well as in *IGHV*-unmutated vs. *IGHV*-mutated patients (*p* < 0.001, Mann–Whitney rank-sum test) (Figure 2D). Association with *TP53* aberrations (*TP53* mutations and/or del17p13) was not observed.

Finally, we observed a different distribution of CLL-IPI risk groups (low, intermediate, high, very high) in IRF2-3^low^ and IRF2-3^high^ patients, with a significantly higher frequency of patients with low CLL-IPI risk scores in the IRF2-3^low^ vs. the IRF2-3^high^ group (*p* < 0.001, Fisher exact test).

The analysis of lnc-KIAA1755-4 expression revealed its positive correlation with WBC and lymphocyte count (r = 0.278, *p* = 0.006 and r = 0.319, *p* = 0.002, respectively; Spearman rank-order correlation test), as well as with serum β2-microglobulin and LDH (r = 0.339, *p* = 0.01 and r = 0.234, *p* = 0.044, respectively; Spearman rank-order correlation test). Conversely, there was a negative correlation with Hb levels (r = −0.289, *p* = 0.004, Spearman rank-order correlation test). No association was detected with age, sex, platelet count and the Binet stage at diagnosis. Among the investigated biological prognostic markers, lnc-KIAA1755-4 exerted a significantly higher expression in *IGHV*-unmutated vs. *IGHV*-mutated patients (*p* = 0.015, Mann–Whitney rank-sum test) (Figure 2E), while the association with CD38 expression, cytogenetic risk and *TP53* aberrations was not found.

In addition, CLL-IPI risk scores were differently distributed in the lnc-KIAA1755-4^low^ and lnc-KIAA1755-4^high^ groups; patients with low CLL-IPI were predominant in the lnc-KIAA1755-4^low^ group, while patients with intermediate, high and very high CLL-IPI were predominant in the lnc-KIAA1755-4^high^ group (*p* = 0.015, Fisher exact test).

Stereotyped BCRs were detected in 15 patients (13.5%), all of which belonged to the “major” stereotyped subsets. The BCR stereotypy was predominantly present in the *IGHV*-unmutated CLL subtype (13/15 patients, 86.7%), and the identified stereotyped BCRs were assigned to subset #1 (five patients), subset #3 (three patients), subset #5 (two patients), subset #31 (one patient), subset #99 (one patient), and subset #202 (one patient). The two stereotyped BCRs detected in the *IGHV*-mutated CLL subtype were assigned to subset #2 (one patient) and subset #4 (one patient). The expression levels of lnc-IRF2-3 and lnc-KIAA1755-4 were not associated with the BCR stereotype either in the whole cohort or in the *IGHV*-unmutated and *IGHV*-mutated groups.

Regarding the pretreatment expression of lnc-IRF2-3 and lnc-KIAA1755-4 in patients with Richter transformation (2/112), we detected a low level of lnc-IRF2-3 and a high level of lnc-KIAA1755-4 in one patient, while the other patient expressed a high level of lnc-IRF2-3 and a low level of lnc-KIAA1755-4.

### 2.3. Prognostic Significance of Lnc-IRF2-3 and Lnc-KIAA1755-4 Expression

Prognostic significance, in terms of the power of predicting TTFT and OS, was first investigated for the two lncRNAs individually.

**Figure 3:**
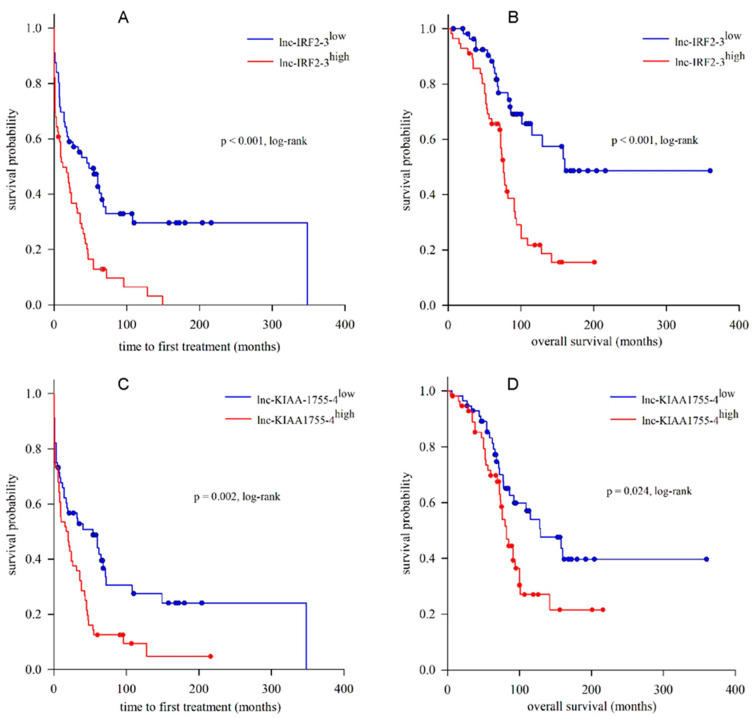
Kaplan–Meier analysis of the time to first treatment (TTFT) and overall survival (OS) in relation to lnc-IRF2-3 and lnc-KIAA1755-4 expression. TTFT and OS were significantly shorter in lnc-IRF2-3^high^ vs. lnc-IRF2-3^low^ group, and in lnc-KIAA1755-4^high^ vs. lnc-KIAA1755-4^low^ group. (**A**) lnc-IRF2-3^low^ (n = 56): median TTFT = 48 months, 95% CI = 19.25–76.78; lnc-IRF2-3^high^ (n = 56): median TTFT = 12 months, 95% CI = 1.27–22.74. (*p* < 0.001, log-rank test). (**B**) lnc-IRF2-3^low^ (n = 56): median OS = 160 months, 95% CI = not determined; lnc-IRF2-3^high^ (n = 56): median OS = 76 months, 95% CI = 69.82–82.18. (*p* < 0.001, log-rank test). (**C**) lnc-KIAA1755-4^low^ (n = 56): median TTFT = 54 months, 95% CI = 10.59–97.413; lnc-KIAA1755-4^high^ (n = 56): median TTFT = 17 months, 95% CI = 2.33–31.67. (*p* = 0.002, log-rank test). (**D**) lnc-KIAA1755-4^low^ (n = 56): median OS = 129 months, 95% CI = 71.87–186.13; lnc-KIAA1755-4^high^ (n = 56): median OS = 82 months, 95% CI = 70.78–93.22. (*p* = 0.024, log-rank test).

Kaplan–Meier analysis revealed that patients classified into the lnc-IRF2-3^high^ group had shorter median TTFT when compared to patients in the lnc-IRF2-3^low^ group (12 and 48 months, respectively; *p* < 0.001, log-rank test) (Figure 3A). Univariate Cox regression analysis confirmed the adverse impact of high lnc-IRF2-3 expression on TTFT (HR = 2.248, 95%CI = 1.456–3.471, *p* < 0.001). In addition, when adjusted for confounding variables, high lnc-IRF2-3 expression was found to predict shorter TTFT independently of CD38 status (HR = 1.977, 95%CI = 1.231–3.174, *p* = 0.005), Binet stage at diagnosis (HR = 1.840, 95%CI = 1.183–2.863, *p* = 0.007) and cytogenetic risk (HR = 1.598, 95%CI = 1.004–2.543, *p* = 0.048). However, after adjustment for *IGHV* SHM status, the predictive power of lnc-IRF2-3 expression was lost (*p* = 0.439). Regarding OS, the lnc-IRF2-3^high^ group had a significantly shorter median OS in comparison to the lnc-IRF2-3^low^ group (76 and 160 months, respectively; *p* < 0.001, log-rank test) (Figure 3B), with an HR of 2.956 (95%CI = 1.687–5.180, *p* < 0.001). Moreover, high lnc-IRF2-3 expression predicted shorter OS independently of CD38 (HR = 3.523, 95%CI = 1.863–6.661, *p* < 0.001), Binet stage (HR = 2.683, 95%CI = 1.449–4.803, *p* = 0.001), cytogenetic risk (HR = 2.274, 95%CI = 1.252–4.132, *p* = 0.007) and *IGHV* SHM status (HR = 2.213, 95%CI = 1.131–4.330, *p* = 0.020). Similarly to lnc-IRF2-3, a comparison of the lnc-KIAA1755-4^high^ and lnc-KIAA1755-4^low^ groups revealed shorter median TTFT in the former group (17 and 54 months, respectively, *p* = 0.002, log-rank test) (Figure 3C). The adverse effect of high lnc-KIAA1755-4 expression on TTFT (HR = 1.905, 95%CI = 1.237–2.935, *p* = 0.003) was independent of CD38 (HR = 1.795, 95%CI = 1.141–2.823, *p* = 0.011), Binet stage (HR = 1.929, 95%CI = 1.254–2.969, *p* = 0.003) and *IGHV* SHM status (HR = 1.638, 95%CI = 1.053–2.547, *p* = 0.028), but not of cytogenetic risk (*p* = 0.090). The negative impact of high lnc-KIAA1755-4 was also detected regarding OS (HR = 1.817, 95%CI = 1.073–3.078, *p* = 0.026), and a significantly shorter median OS was observed in the lnc-KIAA1755-4^high^ vs. lnc-KIAA1755-4^low^ group (82 and 129 months, respectively, *p* = 0.024, log-rank test) (Figure 3D). Taking the confounding variables into account, the predictive power of lnc-KIAA1755-4 expression was shown to be independent of CD38 (HR = 1.939, 95%CI = 1.095–3.435, *p* = 0.023) and Binet stage (HR = 1.764, 95%CI = 1.040–2.991, *p* = 0.035), but was lost after adjustment for *IGHV* SHM status (*p* = 0.114) and cytogenetic risk (*p* = 0.073).

Finally, we evaluated the prognostic significance of combined lnc-IRF2-3 and lnc-KIAA1755-4 expression, i.e., the “2-lncRNA risk model”. In order to do that, we divided our CLL cohort into three groups: (1) “low/low” group, composed of patients with low expression of both lncRNAs; (2) “low/high” group, with discordant expression of lnc-IRF2-3 and lnc-KIAA1755-4; and (3) “high/high” group, in which both lncRNAs were highly expressed.

**Figure 4:**
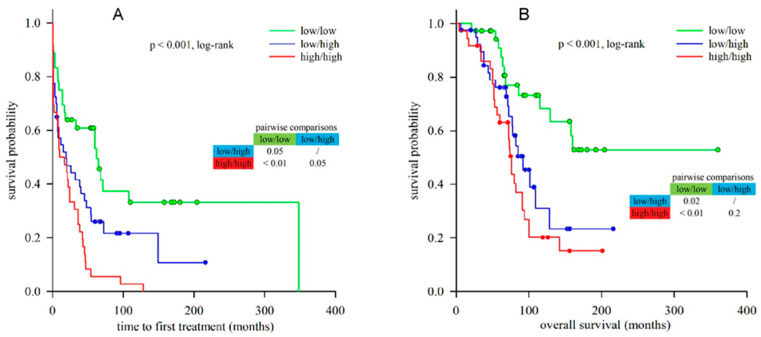
**Kaplan–Meier analysis of TTFT (A) and OS (B) in relation to the combined lnc-IRF2-3 and lnc-KIAA1755-4 expression.** Significant differences in TTFT and OS were observed between CLL patients grouped according to the expression of both lncRNAs. (**A**) “low/low” group (n = 36): median TTFT = 62 months, 95% CI = 30.418–93.582; “low/high” group (n = 40): median TTFT = 19 months, 95% CI = −2.655–40.655; “high/high” group (n = 36): median TTFT = 10 months, 95% CI = −1.760–21.760. (*p* < 0.001, log-rank test). (**B**) “low/low” group (n = 36): median OS not reached; “low/high” group (n = 40): median OS = 92 months, 95% CI = 69.657–114.343; “high/high” group (n = 36): median OS = 76 months, 95% CI = 70.774–81.226. (*p* < 0.001, log-rank test). Low/low and high/high groups are defined by concordant expression of the investigated lncRNAs (lnc-IRF2-3^low^/lnc-KIAA1755-4^low^ and lnc-IRF2-3^high^/lnc-KIAA1755-4^high^, respectively); low/high group is defined by high expression of only one lncRNA (lnc-IRF2-3^low^/lnc-KIAA1755-4^high^ and lnc-IRF2-3^high^/lnc-KIAA1755-4^low^).

Kaplan–Meier analysis showed significant differences in TTFT between these three groups (Figure 4A). The “high/high” group had the shortest median TTFT (10 months) and HR of 3.170 (95%CI = 1.815–5.537, *p* < 0.001), while the median TTFT of the “low/high” group was 19 months and HR 1.186 (95%CI = 1.078–3.301, *p* = 0.026) when compared to the “low/low” group (median TTFT 62 months). However, in multivariate Cox regression analysis controlling for *IGHV* SHM status, CD38, Binet stage and cytogenetic risk, this model lost its predictive power (Table 2). It should be noted that when adjusted for *IGHV* SHM status, CD38, Binet stage and cytogenetic risk separately, the “2-lncRNA risk model” was independent of all covariates except for *IGHV* SHM status (Supplementary Table S2).

When analyzing OS, we observed the shortest median OS in the “high/high” group (76 months) with an HR of 3.620 (95%CI = 1.797–7.292, *p* < 0.001), and a median OS of 92 months with an HR of 2.475 (95%CI = 1.184–5.176, *p* = 0.016) in the “low/high” group, when compared to the “low/low” group (median OS not reached) (Figure 4B). In addition, the “2-lncRNA risk model” was independent of the above-mentioned covariates when they were analyzed separately (Supplementary Table S3). Multivariate Cox regression analysis revealed the independence of the “2-lncRNA risk model” from all four investigated confounders, thus contrasting the findings for TTFT, but pointed to very similar adjusted HRs of the “low/high” and “high/high” groups (Table 2).

Lastly, in our cohort, the “2-lncRNA risk model” was able to predict TTFT and OS independently of CLL-IPI. In both cases, however, the adjusted HRs of the “low/high” and “high/high” groups were closely comparable (Table 2).

## 3. Discussion

Dysregulated expression of lncRNAs was reported in various types of cancer, including B-cell malignancies. By fine-tuning the expression of many genes, lncRNAs are involved in the regulation of both oncogenic and tumor-suppressor pathways, and their aberrant expression has been linked to the pathogenesis, disease progression, metastasis and treatment outcome. However, the role of lncRNAs in the natural history of CLL has been markedly under-researched when compared to other cancers.

In this study, we investigated the expression pattern and prognostic significance of two lncRNAs, lnc-IRF2-3 and lnc-KIAA1755-4, in CLL. Lnc-IRF2-3 is transcribed from an intergenic region located on chromosome 4q35, and has previously been reported to be upregulated in CLL, particularly in the more aggressive, *IGHV*-unmutated subtype [29,45,51]. Interestingly, the opposite was found in plasma cell dyscrasias; lnc-IRF2-3 is being gradually downregulated within the spectrum of these disorders, showing the highest expression in the monoclonal gammopathy of undetermined significance, and the lowest in the most aggressive diseases, i.e., multiple myeloma and plasma cell leukemia [55]. It was shown that lnc-IRF2-3 expression in CLL is positively correlated with the expression of gene sets involved in lipid, amino-acid and sugar metabolism, determination of cell polarity, but also with the gene set associated with the primary immunodeficiency disorders which includes several genes that have pivotal roles in CLL cells’ activation (Bruton tyrosine kinase (*BTK*), *ZAP-70*, *CD40*, *CD79A*, *CD19*, etc.) [29]. However, the mechanisms of lnc-IRF2-3 action have not been elucidated yet.

Lnc-KIAA1755-4 derives from the third intron of the small nucleolar RNA host gene 17 (*SNHG17*; chromosome 20q11-23) and corresponds to SNORA71A, which belongs to the class of small nucleolar non-coding RNAs involved in the post-transcriptional modifications of rRNAs, tRNAs and mRNAs, splicing and also play miRNA-like roles [56]. SNORA71A is aberrantly expressed in various cancers; for example, it is upregulated in colorectal, gallbladder, breast and non-small cell lung cancer, where it enhances proliferation, migration, invasion and drug resistance of malignant cells through targeting different signaling pathways (NF-kappa B, Toll-like receptor, Jak-STAT, AKT/NRF2/GPX4, TGF-β, MAPK/ERK, etc.) [57,58,59,60]. Conversely, in hepatocellular carcinoma, SNORA71A is downregulated, and its low levels are associated with high-risk features and poor survival [61]. In CLL, lnc-KIAA1755-4 was reported to be overexpressed, and positively correlated with the gene sets involved in ribosome formation and translation, transcription and maintenance of chromosomes and telomeres [29]. However, similarly to lnc-IRF2-3, its regulatory functions in CLL cells remained uncharacterized.

We analyzed the expression of lnc-IRF2-3 and lnc-KIAA1755-4 in PBMNC of previously untreated CLL patients and confirmed their overexpression in comparison to healthy individuals. We also observed the association between high expression levels of both lncRNAs and poor-risk prognostic features evidenced at diagnosis. Lnc-IRF2-3 high-expressing patients had higher WBC, lymphocyte counts and serum β2-microglobulin levels than lnc-IRF2-3 low-expressing patients. In addition, high expression of lnc-IRF2-3 was associated with advanced Binet stages, as was reported by El-Khazragy et al. [51]. Furthermore, we observed higher lnc-IRF2-3 expression in patients classified into intermediate and high-risk cytogenetic groups in comparison to patients with low-risk cytogenetics. Previous reports regarding the association of lnc-IRF2-3 expression and chromosomal abnormalities are conflicting; in the study of Ronchetti et al., these associations were not found, while El-Khazragy et al. detected the highest levels of lnc-IRF2-3 in patients with del17p13 [29,51]. Deletion 17p13 encompasses the *TP53* gene, whose aberrations (mutations and/or deletion) are the most adverse prognostic factor in CLL. In our study, the association between *TP53* aberrations and lnc-IRF2-3 expression was not found. Regarding other cellular and molecular prognostic markers, we observed an association between high lnc-IRF2-3 levels with CD38-positivity, which was also reported by Ronchetti et al. (but not by El-Khazragy et al.), as well as with the *IGHV*-unmutated CLL subtype, as was reported in both previous studies [29,51]. It is noteworthy that in the above-mentioned studies, high lnc-IRF2-3 levels were also associated with ZAP-70-positive status and the presence of *NOTCH1* mutations, which are negative prognostic markers in CLL [29,51]. Concerning lnc-KIAA1755-4, we found that its high expression was associated with high WBC, lymphocyte count, serum β2-microglobulin, LDH and low Hb at diagnosis, as well as with the *IGHV*-unmutated status. Expression of both lncRNAs was higher in patients with intermediate, high and very high CLL-IPI risk scores when compared to patients with low CLL-IPI before the initiation of treatment.

The association between lnc-IRF2-3 and lnc-KIAA1755-4 expression and *IGHV* SHM status is intriguing and warrants further research. Ferreira et al. showed that transcriptional landscapes (both coding and non-coding) of *IGHV*-mutated and *IGHV*-unmutated CLL subtypes do not exhibit profound differences [45]. On the other hand, recent studies revealed that *IGHV* SHM status is the main determinant of the coding transcriptome and proteome variability in CLL [62,63]. The prognostic significance of *IGHV* SHM status, along with the restrictions in the immunoglobulin rearrangements’ gene repertoire and B-cell receptor stereotypy, have provided key evidence that BCR (auto)antigenic stimulation drives the leukemogenesis and progression of CLL [64,65]. BCR activation provides survival and proliferative signals for CLL cells, and responsiveness to BCR triggering is higher in *IGHV*-unmutated than in *IGHV*-mutated cells, thus explaining the impact of *IGHV* SHM status on CLL progression and prognosis [66]. LncRNAs were found to undergo major changes in expression during a normal humoral immune response and are highly specific for different stages of this process; some of them are related to the genes activated upon antigen-dependent B cell activation, including those involved in the BCR signaling pathway [16,67]. However, the data about the role of lncRNAs in BCR signaling are scarce. Pyfrom et al. discovered a cytoplasmic, B-cell-specific lncRNA BCALM, involved in intracellular Ca^2+^ signaling upon BCR activation. The authors showed that in diffuse large B-cell lymphoma cells BCALM interacts with kinase adaptor proteins AKAP9 and AKAP1 which form complexes with protein kinases A and C; those kinases activate phospholipase D1 to produce phosphatidic acid which activates SHP-1 phosphatase, a negative regulator of BCR signaling. These results suggest that BCALM promotes the negative feedback loop that down-modulates BCR-mediated Ca^2+^ signaling [41]. In another study that investigated lncRNA expression in chronic graft-versus-host disease, three lncRNAs (NONHSAT142151, NONHSAT040475 and FR118417) were found to strongly correlate with the expression of mRNAs related to the BCR signaling pathway (BTK, CD72, DAPP1, LILRB3, NFKBIE, RASGRP3). The functional relevance of those lncRNAs, though, remains unclear [68]. Bearing in mind the above-mentioned findings, it is plausible that lncRNAs are involved in the regulation of the BCR pathway in CLL as well. Regarding the two lncRNAs investigated in our study, the data about their potential relationship with BCR signaling in CLL are lacking, except the positive correlation of lnc-IRF2-3 expression with the expression of *ZAP-70* and *BTK* reported by Ronchetti et. al. and El-Khazragy et. al [29,51].

Our results revealed that high expression levels of lnc-IRF2-3 and lnc-KIAA1755-4 were associated with poor survival of CLL patients in our cohort. Patients belonging to the lnc-IRF2-3^high^ group had significantly shorter median TTFT and OS in comparison to patients in the lnc-IRF2-3^low^ group. The predictive power of lnc-IRF2-3 expression for TTFT was independent of Binet stage, CD38 status and cytogenetic risk, but not of *IGHV* SHM status, which is probably due to the large overlap between lnc-IRF2-3^high^ and *IGHV*-unmutated groups of patients. The same was found for the predictive power for OS, except that it was also independent of *IGHV* SHM status. Similarly to lnc-IRF2-3, high lnc-KIAA1755-4 expression was also associated with shorter median TTFT and OS. Its predictive power for TTFT was found to be independent of Binet stage, CD38 status and *IGHV* SHM status (but not of cytogenetic risk), whereas the adverse effect on OS was independent only of Binet stage and CD38 status. Concerning the associations with OS that we observed, it should be underlined that the majority of our patients underwent treatment during the observational period of our study, predominantly with FC-based regimens. However, we detected significantly shorter OS in patients with high lnc-IRF2-3 expression as was reported in the study of El-Khazragy et al., although the cohort analyzed in that study did not include patients who received any treatment, and the follow-up time was considerably shorter than in our study [51]. Since there are no data about the effect of chemo(immuno)therapy on the expression of lnc-IRF2-3, as well as about the possible influence of lnc-IRF2-3 on the response to treatment, the similarities between the results obtained in ours and in the study of El-Khazragy et al. regarding OS remain to be explained. Of note, the association of lnc-KIAA1755-4 expression with OS was not investigated previously.

In order to test the “2-lncRNA risk model”, we applied the same approach as described in Ronchetti et al. and, by combining the expression levels of lnc-IRF2-3 and lnc-KIAA1755-4, defined three patient groups: “low/low” and “high/high”, with the concordant expression of both lncRNAs, and “low/high”, with high expression of one of the two lncRNAs [29]. The three risk groups had significantly different median TTFT in our cohort, with the shortest TTFT in the “high/high” group, as predicted by the “2-lncRNA risk model”. However, in multivariate analysis controlling for Binet stage, CD38 status, cytogenetic risk and *IGHV* SHM status, the model lost its predictive power. This contrasts the findings of Ronchetti et al. in whose study the “2-lncRNA risk model” was able to predict TTFT independently of *IGHV* SHM status, CD38 and ZAP-70 expression, *NOTCH1* mutations and unfavorable chromosomal aberrations (trisomy 12, del11q22-23, del17p13) [29]. The discrepancy between the results of our two studies concerning this issue may be attributed to the fact that the cohort analyzed in the study of Ronchetti et al. consisted solely of Binet A patients (whereas ours was heterogeneous regarding Binet stages), as well as to the different confounding variables used in multivariate analyses [29]. In addition, we analyzed the power of the “2-lncRNA risk model” for predicting OS and, again, the shortest OS was observed in the “high/high” group. Moreover, in multivariate analysis, the model remained significant and independent of Binet stage, CD38 status, cytogenetic risk and *IGHV* SHM status. However, the adjusted hazard ratios of the “low/high” and “high/high” groups were very similar, suggesting that high expression of at least one of the two lncRNAs is sufficient for OS significantly shorter than OS when both lncRNAs are expressed at low levels. Interestingly, in our cohort, the “2-lncRNA risk model” was associated with both TTFT and OS independently of CLL-IPI, despite the fact that lnc-IRF2-3 and lnc-KIAA1755-4 expression levels were strongly associated with three out of five variables incorporated into the CLL-IPI stratification model (Binet stage, serum β2-microglobulin and *IGHV* SHM status) [69]. In the study of Ronchetti et al., the power of the “2-lncRNA risk model” to predict TTFT was independent of the Progression–Risk Score, which integrates clinical and genetic variables similar to CLL-IPI (Rai stage, absolute lymphocyte count, serum β2-microglobulin and *IGHV* SHM status) [29,70]. However, in our study the hazard ratios of the “low/high” and “high/high” groups adjusted for CLL-IPI were very similar (in both TTFT and OS prediction), unlike the HRs adjusted for PRS reported by Ronchetti et al. Given that the number of patients for whom the CLL-IPI scores were available was relatively small, our findings should be confirmed in larger cohorts. Currently, in the absence of information about the pathways in which lnc-IRF2-3 and lnc-KIAA1755-4 participate in CLL cells, as well as their potential interactions, it is hard to speculate whether the two lncRNAs affect TTFT and OS synergistically or independently of each other.

In conclusion, the results of our study show that high expression levels of lnc-IRF2-3 and lnc-KIAA1755-4 at diagnosis are markers of poor prognosis in CLL. However, the exact roles that those two lncRNAs play during the pathogenesis and/or progression of the disease remain to be elucidated. Our finding that the expressions of lnc-IRF2-3 and lnc-KIAA1755-4 are positively correlated in CLL but not in normal samples implies that maybe a common mechanism underlies their overexpression in CLL. Given that lncRNAs both regulate and are regulated by oncogenes and tumor-suppressor genes, aberrant expression of lnc-IRF2-3 and lnc-KIAA1755-4 might be involved in the malignant transformation of the CLL cell of origin or may be its consequence. In any case, the mechanism(s) of their dysregulation are currently unknown. Besides transcriptional regulation by transcription factors (e.g., Myc, p53), the mechanisms involved in lncRNA aberrant expression in cancer include copy number alterations, mutations, epigenetic changes at lncRNA loci, RNA-processing abnormalities, Epstein–Barr virus infection, etc. [15,71]. Thus, the causes of lnc-IRF2-3 and lnc-KIAA1755-4 upregulation in CLL may be complex, and their deciphering could point to the pathways in which the two lncRNAs are engaged. It is also important to investigate whether the expression of lnc-IRF2-3 and lnc-KIAA1755-4 changes during the progression of CLL. The observed higher expression of the two lncRNAs in more advanced clinical stages suggests that this might be the case, but it should be confirmed by analyzing the expression patterns in individual patients during their disease course. In addition, the possibility that lnc-IRF2-3 and lnc-KIAA1755-4 levels influence the response to treatment should also be explored. To resolve all these issues, future mechanistic studies should be directed toward identifying the regulatory networks and target specificities of lnc-IRF2-3 and lnc-KIAA1755-4 in CLL cells. A better understanding of the functional roles that lnc-IRF2-3 and lnc-KIAA1755-4 play in the complex pathobiology of CLL cells will be crucial for evaluating their clinical utility as prognostic and/or predictive molecular markers and potential therapeutic targets.

## 4. Materials and Methods

### 4.1. Study Cohort

A total of 112 unselected CLL patients, diagnosed, treated and followed at the Clinic for Hematology, University Clinical Center of Serbia (Belgrade, Serbia) from 1993 to 2023, and 21 healthy controls, were enrolled in this study. The diagnosis of CLL was established according to the guidelines of the International Workshop on Chronic Lymphocytic Leukemia (IwCLL) [1].

All the analyses were performed on samples collected prior to initiation of therapy. Clinical and laboratory characteristics of patients were evaluated at diagnosis, while molecular and cytogenetics markers were determined either at diagnosis or during the period from diagnosis to the first treatment.

### 4.2. Analytical Methods

Quantification of lnc-IRF2-3 and lnc-KIAA1755-4 in PBMNC of CLL patients and healthy controls was performed by qRT-PCR. Mononuclear cells were isolated from peripheral blood samples by Ficoll-Paque Plus (GE Healthcare, Chicago, IL, USA) density-gradient centrifugation and total cellular RNA was extracted using TRI reagent (ThermoFisher Scientific, Waltham, MA, USA). RNA was reverse-transcribed using RevertAid M-MuLV Reverse Transcriptase (ThermoFisher Scientific, Waltham, MA, USA) and random hexamer primers according to the manufacturer’s instructions. qRT-PCR amplification of target lncRNAs was carried out using SYBR Green chemistry, in 7900 HT Fast Real-Time PCR system (ThermoFisher Scientific, Waltham, MA, USA). The specific primers designed by Ronchetti et al. were used in qRT-PCR reactions (lnc-IRF2-3 forward 5′-GCAAAGGACCAAGAAAGCTG-3′; lnc-IRF2-3 reverse 5′-CATACACAGGAGGCCTGGAT-3′; lnc-KIAA1755-4 forward 5′-CTCCTGCATCCGAAAGTGAT-3′; lnc-KIAA1755-4 reverse 5′-ATAGGGTGGACCCTCCAAAC-3′) [29]. The reaction mixtures contained 50 ng cDNK, 1 × Power SYBR^®^ Green PCR Master Mix (ThermoFisher Scientific, Waltham, MA, USA) and 3 pmol of each gene-specific primer, in a final reaction volume of 10 µL. The cycling conditions were as follows: denaturation of the template at 95 °C for 10 min, followed by 40 cycles of 95 °C for 15 s and 60 °C for 1 min. All qRT-PCR reactions were run in duplicate, in order to evaluate reproducibility of the results. Relative expression of target lncRNAs was quantified by comparative ddCt method, using glyceraldehyde phosphate dehydrogenase (GAPDH) as endogenous control gene and healthy controls as the calibrator (ddCt = dCt_sample_ − dCt_healthy controls (median)_). The results were expressed in terms of relative units (RU).

The percentage of CLL cells and surface CD38 expression were determined by flow cytometry [72,73]. Common CLL-associated chromosomal aberrations (del13q14, trisomy 12, del11q22-23 and del17p13) were detected by fluorescence in situ hybridization (FISH) on interphase nuclei obtained from peripheral blood, using a panel of locus-specific probes (Vysis/Abbott Laboratories, Abbot Park, IL, USA) according to the manufacturer’s instructions. *IGHV* SHM status, BCR stereotypy and *TP53* mutations were analyzed by Sanger sequencing as recommended by the European Research Initiative on CLL (ERIC) [74,75]. CLL-IPI scores were calculated as recommended by the International CLL-IPI Working Group [69].

### 4.3. Statistical Analysis

Categorical variables are presented as absolute numbers and frequencies, while continuous variables are presented using medians with ranges, 95% CI, and 25th and 75th percentiles. The relationships between categorical variables were analyzed using Fisher’s exact test. The data distribution of continuous variables was assessed using the Shapiro–Wilk test, and they were analyzed using the Student *t*-test and Mann–Whitney rank-sum test. The correlation between continuous variables was analyzed using the Pearson product–moment correlation test and Spearman rank-order correlation test.

TTFT was defined as the time from diagnosis to the first therapy line. OS was defined as the time of diagnosis to the time of death from any cause or last follow-up. Survival analyses were conducted using the Kaplan–Meier method and the log-rank test, as well as with univariate and multivariate Cox regression. The results of Cox regression analyses are presented as the hazard ratio (HR) with 95% CI.

Statistical analysis was performed using SPSS 21.0 software (IBM). All calculated *p*-values were two-tailed and significance was defined as *p* < 0.05.

## Figures and Tables

**Table 1:** CLL patients’ characteristics and their relationship with the expression of lnc-IRF2-3 and lnc-KIAA1755-4.

Variable	Whole Cohort (n = 112)	lnc-IRF2-3^low^ Group (n = 56)	lnc-IRF2-3^high^ Group (n = 56)	*p*-Value	lnc-KIAA1755-4^low^ Group (n = 56)	lnc-KIAA1755-4^high^ Group (n = 56)	*p*-Value
**Sex** (n = 112) ^a^, male/female	79/33	39/17	40/16	1.000 ^d^	36/20	43/13	0.213 ^d^
**Age** (years) (n = 111) ^a^, median (range)	59 (33–80)	60 (33–80)	58.5 (38–75)	0.127 ^e^	59.5 (33–80)	59 (38–75)	0.593 ^e^
**WBC [×10^9^/L]**, (n = 98) ^a^, median (range)	38.6 (6.8–570)	26.4 (6.8–570)	46.2 (10.1–476)	**0.005 ^f^**	29.6 (6.8–476)	46 (8.4–570)	**0.038 ^f^**
**Ly [×10^9^/L]**, (n = 89) ^a^, median (range)	29 (2.7–558.6)	19.6 (2.7–558.6)	39 (6.6–447.4)	**0.004 ^f^**	20.2 (2.7–447.4)	39.1 (4.7–558.6)	**0.013 ^f^**
**Platelets [×10^9^/L]**, (n = 96) ^a^, median (range)	173.5 (1–432)	175 (4–320)	172.5 (1–432)	0.540 ^e^	171 (3–361)	174 (1–432)	0.872 ^e^
**Hb [g/L]**, (n = 97) ^a^, median (range)	138 (44–178)	138.5 (84–178)	138 (44–173)	0.382 ^f^	144 (84–178)	130.5 (44–163)	**0.004 ^f^**
**β2M [mg/L]** (n = 57) ^a^, median (range)	3.2 (0.2–11.5)	2.5 (0.2–11.5)	3.6 (2–11.3)	**0.017 ^f^**	2.5 (0.2–8.7)	3.9 (1.5–11.5)	**0.012 ^f^**
**LDH level** ^b^, (n = 75) ^a^, median (range)	1.4 (0.3–4.2)	1.3 (0.5–3.7)	1.5 (0.3–4.2)	0.424 ^f^	1.2 (0.3–3.6)	1.5 (0.5–4.2)	0.064 ^f^
**Binet stage** (n = 111) ^a^, n (%)				**0.011 ^d^**			0.465 ^d^
A	52 (46.9)	32 (57.1)	20 (36.4)		26 (46.4)	26 (47.3)	
B	43 (38.7)	14 (25)	29 (52.7)		24 (42.9)	19 (34.5)	
C	16 (14.4)	10 (17.9)	6 (10.9)		6 (10.7)	10 (18.2)	
**CD38 status** (n = 101) ^a^, n (%)				0.082 ^d^			0.277 ^d^
positive (≥30%)	30 (29.7)	10 (20.8)	20 (37.7)		12 (24)	18 (35.3)	
negative (˂30%)	71 (70.3)	38 (79.2)	33 (62.3)		38 (76)	33 (64.7)	
**Cytogenetic risk** (n = 97) ^a^, n (%)				**0.007 ^d^**			0.352 ^d^
favorable (del13q14) ^c^	31 (32)	21 (46.7)	10 (19.2)		17 (37.8)	14 (26.9)	
intermediate (no aberrations, trisomy 12)	42 (43.3)	13 (28.9)	29 (55.8)		16 (35.5)	26 (50)	
unfavorable (del11q22-23, del17p13)	24 (24.7)	11 (24.4)	13 (25)		12 (26.7)	12 (23.1)	
***TP53* mutational status** (n = 60) ^a^, n (%)				0.736 ^d^			0.612 ^d^
wt	49 (81.7)	17 (85)	32 (80)		19 (65.5)	30 (71.4)	
mutated	11 (18.3)	3 (15)	8 (20)		10 (34.5)	12 (28.6)	
***IGHV* SHM status** (n = 112) ^a^, n (%)				**<0.001** **^d^**			**0.034 ^d^**
mutated	46 (41.1)	39 (69.6)	7 (12.5)		29 (51.8)	17 (30.4)	
unmutated	66 (58.9)	17 (30.4)	49 (87.5)		27 (48.2)	39 (69.6)	
**CLL-IPI** (n = 53) ^a^, n (%)				**0.004 ^d^**			**0.031 ^d^**
low risk (score 0–1)	8 (15.1)	8 (33.3)	0 (0)		7 (30.4)	1 (3.3)	
intermediate risk (score 2–3)	18 (34)	6 (25)	12 (41.4)		8 (34.8)	10 (33.3)	
high risk (score 4–6)	22 (41.5)	9 (37.5)	13 (44.8)		6 (26.1)	16 (53.3)	
very high risk (score 6–10)	5 (9.4)	1 (4.2)	4 (13.8)		2 (8.7)	3 (10)	

Abbreviations: WBC = white blood cells; Ly = lymphocytes; Hb = hemoglobin; β2M = β2-microglobulin; LDH = lactate dehydrogenase; *IGHV* SHM status = somatic hypermutational status of immunoglobulin heavy variable genes; wt = wild-type; CLL-IPI, The International Prognostic Index for CLL; n = number of patients. ^a^ total number of patients for whom the values of the given parameter at diagnosis/prior to the first therapy line were available. ^b^ LDH level was calculated for each patient as LDH [U/L]/upper limit of normal [U/L]. ^c^ deletion 13q14 occurring as a sole chromosomal aberration. ^d^ Fisher exact test. ^e^ *t*-test. ^f^ Mann–Whitney rank-sum test.

**Table 2:** Multivarate Cox regression analysis of variables associated with TTFT and OS.

Covariates ^*^	TTFT				OS		
	HR	95% CI	*p*-Value		HR	95% CI	*p*-Value
**lnc-IRF2-3 and lnc-KIAA1755-4 expression** ** (low/low vs. low/high)**	1.416	0.734–2.733	0.300		3.538	1.394–8.981	**0.008**
**lnc-IRF2-3 and lnc-KIAA1755-4 expression** ** (low/low vs. high/high)**	1.304	0.640–2.657	0.465		3.866	1.484–10.074	**0.006**
**Binet stage** (A vs. B + C)	2.371	1.461–3.848	<0.001		1.263	0.711–2.245	0.426
**CD38 status** (positive vs. negative)	1.035	0.629–1.702	0.893		1.138	0.644–2.010	0.657
**cytogenetic risk** (favorable vs. intermediate + unfavorable)	1.331	0.743–2.384	0.336		0.987	0.488–1.996	0.970
***IGHV* SHM status** (mutated vs. unmutated)	1.713	0.916–3.202	0.092		1.236	0.588–2.600	0.576
**lnc-IRF2-3 and lnc-KIAA1755-4 expression** ** (low/low vs. low/high)**	2.581	1.021–6.526	**0.045**		7.016	2.028–24.268	**0.002**
**lnc-IRF2-3 and lnc-KIAA1755-4 expression** **(low/low vs. high/high)**	3.195	1.289–7.923	**0.012**		6.165	1.846–20.591	**0.003**
**CLL-IPI** (low vs. intermediate + high + very high)	4.188	0.90–19.490	0.068		0.323	0.089–1.167	0.085

Abbreviations: HR = hazard ratio; CI = confidence interval. ^*^ the first category in brackets was considered as reference.

## Data Availability

The data that support the findings of this study are available from the corresponding author upon request.

## Supplementary Materials

The following supporting information can be downloaded at: https://www.mdpi.com/article/10.3390/ijms26031153/s1.
