# Correction: Tosic et al. High Expression Levels of the Long Non-Coding RNAs Lnc-IRF2-3 and Lnc-KIAA1755-4 Are Markers of Poor Prognosis in Chronic Lymphocytic Leukemia. *Int. J. Mol. Sci.* 2025, *26*, 1153

**DOI:** 10.3390/ijms27010192

**Published:** 2025-12-24

**Authors:** Natasa Tosic, Kristina Tomic Vujovic, Vojin Vukovic, Nikola Kotur, Biljana Stankovic, Irena Marjanovic, Darko Antic, Sofija Sarac, Tamara Bibic, Jelena Ivanovic, Branka Zukic, Teodora Karan-Djurasevic

**Affiliations:** 1Institute of Molecular Genetics and Genetic Engineering, University of Belgrade, Vojvode Stepe 444a, 11042 Belgrade, Serbia; natasa.tosic@imgge.bg.ac.rs (N.T.); nikola.kotur@imgge.bg.ac.rs (N.K.); biljana.stankovic@imgge.bg.ac.rs (B.S.); irena.marjanovic@imgge.bg.ac.rs (I.M.); branka.zukic@imgge.bg.ac.rs (B.Z.); 2Clinic for Hematology, University Clinical Center of Serbia, 11000 Belgrade, Serbia; kristinat992@gmail.com (K.T.V.); vojinvukovic@yahoo.com (V.V.); darko.antic1510976@gmail.com (D.A.); sofijasarac@gmail.com (S.S.); tmedic83@gmail.com (T.B.); jivanovic09@gmail.com (J.I.); 3School of Medicine, University of Belgrade, 11000 Belgrade, Serbia

## References

The following references [24,26] have been retracted and should be removed from the original publication [1]:

24.Cheng, H.; Yan, Z.; Wang, X.; Cao, J.; Chen, W.; Qi, K.; Zhou, D.; Xia, J.; Qi, N.; Li, Z.; et al. Downregulation of long non-coding RNA TUG1 suppresses tumor growth by promoting ubiquitination of MET in diffuse large B-cell lymphoma. *Mol. Cell. Biochem*. **2019**, *461*, 47–56.26.Zhao, X.; Tian, X. Knockdown of long noncoding RNA HOTAIR inhibits cell growth of human lymphoma cells by upregulation of miR-148b. *J. Cell. Biochem*. **2019**, *120*, 12348–12359.

With this correction, the order of some references has been adjusted accordingly.

In order to maintain accuracy and precision, a correction has been made to the sentence containing the original references [24,26] in Section 1, Introduction, Paragraph Number 3:

LncRNAs also modulate other signaling pathways related to apoptosis and proliferation, e.g., PI3K/Akt (HOTAIR), MAPK/Erk (PANDA), NF-κB (DLEU1/2), Wnt/β-catenin (OR3A4, FIRRE, SMAD5-AS1), Il6 (lnc-TOMM7-1), and regulate the expression of cell cycle checkpoint proteins (MALAT1, lincRNA-p21, LUNAR1, MINCR) [23–33].

The authors state that the scientific conclusions are unaffected. The corrections were approved by the Academic Editor. The original publication has also been updated.
