# Supplementary material for: High Expression Levels of the Long Non-Coding RNAs Lnc-IRF2-3 and Lnc-KIAA1755-4 Are Markers of Poor Prognosis in Chronic Lymphocytic Leukemia
Source: Int J Mol Sci. 2025 Jan 29;26(3):1153. doi: 10.3390/ijms26031153 (PMC11817519; doi:10.3390/ijms26031153)
Supplement: Supplementary file 1 [file ijms-26-01153-s001.zip › Supplementary Table S1.pdf]

Supplementary Table S1.Therapeutic regimens used in the first treatment line

Regimen	Dose	Schedule	Number of treated patients
FC(R)		Every 4 weeks for up to 6 courses	
Rituximab	375mg/m^2 in Icy	D1	
	500mg/m^2 in II-VIcy	D1	
Fludarabine	25mg/m^2	D2-D4 (D1-D3 in FC)	
Cyclophoshamide	250mg /m^2	D2-D4 (D1-D3 in FC)	67 (75.3%)*
FC(R)-Lite		Every 4 weeks for up to 6 courses	
Rituximab	375mg/m^2 in Icy, 500mg/m^2 in II-VIcy	D1	
Fludarabine	20mg/m^2	D2-D4 (D1-D3 in FC-Lite)	
Cyclophoshamide	150mg/m^2	D2-D4 (D1-D3 in FC-Lite)	
COP		Every 3 weeks for up to 6-8 courses	
Cyclophoshamide	750mg/m^2	D1	
Vincristine	1.4mg/m^2 (max. 2mg)	D1	7 (8%)
Prednisone	100mg daily	D1-D5	
Chlorambucil	10mg	D1-D7 every 4 week for up to 12 cycles	7 (8%)
Obinutuzumab + chlorambucil		Every 4 weeks for 6 courses	
Obinutuzumab	100mg	D1 Icy	
	900mg	D2 Icy	
	1000mg	D8, D15 Icy, D1 in II-VIcy	3 (3.3%)
Chlorambucil	0.5mg/kg	D1 and D15	
(R) Bendamustin		Every 4 weeks for up to 6 courses	
Rituximab	375mg/m^2 in Icy	D1	
	500mg/m^2 in II-VIcy	D1	1 (1.1%)
Bendamustin	90mg/m^2	D2-D3 (D1-D2 in Bendamustin monotherapy)	
Alemtuzumab (Campath)		1 week	
	3mg	Wednesday	
	10mg	Thursday	
	30mg	Friday	1 (1.1%)
	30mg	2-12 weeks (Mondays, Wednesdays, Fridays)	
Ibrutinib	420mg once daily orally	Continuous treatment	2 (2.2%)
Splenectomy			1 (1.1%)

FCR – fludarabine, cyclophosphamide, rituximab, FC – fludarabine, cyclophosphamide, COP – cyclophosphamide, vincristine, prednisone, cy – cycle, D – day

*among patients treated with FC based regimen, 44 (65.7%) received FC, while 23 (34.3%) FCR/FCR-lite
