# Supplementary material for: High Expression Levels of the Long Non-Coding RNAs Lnc-IRF2-3 and Lnc-KIAA1755-4 Are Markers of Poor Prognosis in Chronic Lymphocytic Leukemia
Source: Int J Mol Sci. 2025 Jan 29;26(3):1153. doi: 10.3390/ijms26031153 (PMC11817519; doi:10.3390/ijms26031153)
Supplement: Supplementary file 1 [file ijms-26-01153-s001.zip › Supplementary table S2.pdf]

Supplementary Table S2. Cox regression analysis of lncRNAs' predictive power for TTFT, controlling for Binet Stage (A), CD38 status (B), cytogenetic risk (C) and *IGHV* SHM status (D)

**A** covariates	**TTFT**		
	**HR**	**95% CI**	**p-value**
**lnc-IRF2-3 and lnc-KIAA1755-4 expression (low/low vs. low/high)**	1.802	1.041-3.190	0.036
**lnc-IRF2-3 and lnc-KIAA1755-4 expression (low/low vs. high/high)**	2.686	1.538-4.690	0.001
**Binet stage** (A vs. B+C)	2.837	1.806-4.458	0.001

**B** covariates	**TTFT**		
	**HR**	**95% CI**	**p-value**
**lnc-IRF2-3 and lnc-KIAA1755-4 expression (low/low vs. low/high)**	1.679	0.929-3.035	0.086
**lnc-IRF2-3 and lnc-KIAA1755-4 expression (low/low vs. high/high)**	2.738	1.511-4.964	0.001
**CD38 status** (positive vs. negative)	1.683	1.056-2.681	0.029

**C** covariates	**TTFT**		
	**HR**	**95% CI**	**p-value**
**lnc-IRF2-3 and lnc-KIAA1755-4 expression (low/low vs. low/high)**	1.862	1.046-3.315	0.035
**lnc-IRF2-3 and lnc-KIAA1755-4 expression (low/low vs. high/high)**	2.004	1.110-3.616	0.021
**cytogenetic risk** **(**favorable vs. intermediate + unfavorable**)**	1.715	1.015-2.899	0.044

**D** covariates	**TTFT**		
	**HR**	**95% CI**	**p-value**
**lnc-IRF2-3 and lnc-KIAA1755-4 expression (low/low vs. low/high)**	1.441	0.806-2.579	0.218
**lnc-IRF2-3 and lnc-KIAA1755-4 expression (low/low vs. high/high)**	1.857	0.995-3.466	0.052
***IGHV* SHM status** (mutated vs. unmutated)	2.556	1.497-4.364	0.001

Abbreviations: HR = hazard ratio; CI = confidence interval

The first category in brackets was considered as reference.
Low/low and high/high groups are defined by concordant expression of the investigated lncRNAs (lnc-IRF2-3^{low}/lnc-KIAA1755-4^{low} and lnc-IRF2-3^{high}/lnc-KIAA1755-4^{high}, respectively); low/high group is defined by high expression of only one lncRNA (lnc-IRF2-3^{low}/lnc-KIAA1755-4^{high} and lnc-IRF2-3^{high}/lnc-KIAA1755-4^{low})
