# Supplementary material for: High Expression Levels of the Long Non-Coding RNAs Lnc-IRF2-3 and Lnc-KIAA1755-4 Are Markers of Poor Prognosis in Chronic Lymphocytic Leukemia
Source: Int J Mol Sci. 2025 Jan 29;26(3):1153. doi: 10.3390/ijms26031153 (PMC11817519; doi:10.3390/ijms26031153)
Supplement: Supplementary file 1 [file ijms-26-01153-s001.zip › Supplementary table S3.pdf]

Supplementary Table S3. Cox regression analysis of lncRNAs' predictive power for OS, controlling for Binet Stage (A), CD38 status (B), cytogenetic risk (C) and *IGHV* SHM status (D)

**A**<br>**covariates**	**OS**		
	**HR**	**95% CI**	**p-value**
**lnc-IRF2-3 and lnc-KIAA1755-4 expression (low/low vs. low/high)**	2.367	1.131-4.957	0.022
**lnc-IRF2-3 and lnc-KIAA1755-4 expression (low/low vs. high/high)**	3.251	1.594-6.629	0.001
**Binet stage** (A vs. B+C)	1.482	0.865-2.538	0.152

**B**<br>**covariates** [a]	**OS**		
	**HR**	**95% CI**	**p-value**
**lnc-IRF2-3 and lnc-KIAA1755-4 expression (low/low vs. low/high)**	3.793	1.565-9.192	0.003
**lnc-IRF2-3 and lnc-KIAA1755-4 expression (low/low vs. high/high)**	4.917	2.102-11.504	< 0.001
**CD38 status** (positive vs. negative)	1.403	0.803-2.451	0.235

**C**<br>**covariates** [a]	**OS**		
	**HR**	**95% CI**	**p-value**
**lnc-IRF2-3 and lnc-KIAA1755-4 expression (low/low vs. low/high)**	2.239	1.060-4.727	0.035
**lnc-IRF2-3 and lnc-KIAA1755-4 expression (low/low vs. high/high)**	2.761	1.337-5.702	0.006
**cytogenetic risk** (favorable vs. intermediate + unfavorable)	1.472	0.781-2.777	0.232

**D**<br>**covariates** [a]	**OS**		
	**HR**	**95% CI**	**p-value**
**lnc-IRF2-3 and lnc-KIAA1755-4 expression (low/low vs. low/high)**	2.180	1.027-4.627	0.043
**lnc-IRF2-3 and lnc-KIAA1755-4 expression (low/low vs. high/high)**	2.565	1.186-5.548	0.017
***IGHV* SHM status** (mutated vs. unmutated)	1.929	1.016-3.662	0.045

Abbreviations: HR = hazard ratio; CI = confidence interval

The first category in brackets was considered as reference.
Low/low and high/high groups are defined by concordant expression of the investigated lncRNAs (lnc-IRF2-3^{low}/lnc-KIAA1755-4^{low} and lnc-IRF2-3^{high}/lnc-KIAA1755-4^{high}, respectively); low/high group is defined by high expression of only one lncRNA (lnc-IRF2-3^{low}/lnc-KIAA1755-4^{high} and lnc-IRF2-3^{high}/lnc-KIAA1755-4^{low})
